# Induced Pluripotent Stem Cell-Based Cancer Vaccines

**DOI:** 10.3389/fimmu.2019.01510

**Published:** 2019-07-08

**Authors:** Xiaoming Ouyang, Melinda L. Telli, Joseph C. Wu

**Affiliations:** ^1^Cardiovascular Institute, School of Medicine, Stanford University, Stanford, CA, United States; ^2^Institute for Stem Cell Biology and Regenerative Medicine, School of Medicine, Stanford University, Stanford, CA, United States; ^3^Department of Medicine, Stanford University, Stanford, CA, United States; ^4^Department of Radiology, Stanford University, Stanford, CA, United States

**Keywords:** cancer vaccine, iPSC, ESC, oncofetal antigen, cancer stem cell

## Abstract

Over a century ago, it was reported that immunization with embryonic/fetal tissue could lead to the rejection of transplanted tumors in animals. Subsequent studies demonstrated that vaccination of embryonic materials in animals induced cellular and humoral immunity against transplantable tumors and carcinogen-induced tumors. Therefore, it has been hypothesized that the shared antigens between tumors and embryonic/fetal tissues (oncofetal antigens) are the key to anti-tumor immune responses in these studies. However, early oncofetal antigen-based cancer vaccines usually utilize xenogeneic or allogeneic embryonic stem cells or tissues, making it difficult to tease apart the anti-tumor immunity elicited by the oncofetal antigens vs. graft-vs.-host responses. Recently, one oncofetal antigen-based cancer vaccine using autologous induced pluripotent stem cells (iPSCs) demonstrated marked prophylactic and therapeutic potential, suggesting critical roles of oncofetal antigens in inducing anti-tumor immunity. In this review, we present an overview of recent studies in the field of oncofetal antigen-based cancer vaccines, including single peptide-based cancer vaccines, embryonic stem cell (ESC)- and iPSC-based whole-cell vaccines, and provide insights on future directions.

## Introduction

Cancer cells have the capability to proliferate indefinitely and metastasize to different parts of the body. Embryonic stem cells (ESCs) have the ability to undergo rapid clonal proliferation and self-renew, and can inhabit and thrive in various environments of the human body. The similarities between fetal development and cancer have long been recognized (1) following the discovery of oncofetal proteins and antigens such as α-fetoprotein (AFP) (2), carcinoembryonic antigen (CEA) (3), and human chorionic gonadotropic (HCG) (4) (Supplemental Table 1). These proteins are tumor associated proteins or antigens (TAA) that are synthesized during embryonic development and appear again in adults during cancer development. Furthermore, these proteins are well-known biomarkers for cancer detection and monitoring (3, 5–9). Induced pluripotent stem cells (iPSCs) can be generated by introducing four transcription factors into adult somatic cells, which transform their transcriptional and epigenetic state to a pluripotent one that closely resembles ESCs (10). Similar to ESCs, iPSCs share genetic and transcriptomic signatures with cancer cells, including protein markers that can be recognized by the immune system (11, 12).

Schöne recognized over a century ago that immunization with embryonic/fetal tissue could lead to the rejection of transplanted tumors in animals (13). Later studies indicated that vaccination of embryonic materials in animals elicited humoral and cellular immunity against transplantable tumors and carcinogen-induced tumors, supporting the idea that anti-tumor immunity may arise from the antigens shared between fetal tissue and cancer cells. Recent studies provided evidence that oncofetal antigen-based cancer vaccines could elicit potent T cell responses (5–9). However, there are problems associated with utilizing embryonic/fetal materials for the development of anti-cancer vaccines. Ethical issues, tumorigenicity, and alloimmunity have been the main limitations of using ESCs for clinical applications. Therefore, a substitute for ESCs is needed for overcoming these obstacles. A recent study using an irradiated autologous iPSC-based cancer vaccine has started to address these issues (14). Moreover, the use of ESCs/iPSCs alone as an anti-cancer vaccine only showed moderate anti-tumor effects in some of the early studies (13, 15, 16), suggesting that vaccine adjuvants may be needed in combination with ESCs/iPSCs to enhance innate immunity and increase antigen presentation. Here, we summarize and compare recent studies in addressing these challenges.

## Cancer Cells Are Remarkably Similar to ESCS and iPSCs

Cancer cells and ESCs share many cellular and molecular features. These include a rapid proliferation rate (17), upregulated activity of telomerase (18), increased expression levels of oncogenes such as c-*MYC* (19) and krupple-like factor 4 (*KLF4*) (20), and similar overall gene expression profiles (21, 22), microRNA signatures (23), and epigenetic status (24). Similar to cancer cells, after long-term culture the ESC lines will continue to proliferate actively and express high levels of telomerase activity, allowing them to maintain telomere length and cellular immortality (18, 25, 26). These features of ESCs resemble the hallmarks of cancer cells that have “sustaining proliferative signaling” and “replicative immortality” (27).

The discovery of iPSCs in 2006 (10, 28) has revolutionized the field of stem cell research. Human iPSCs reprogrammed from a patient's somatic tissues share almost the same gene expression profiles with that patient's ESCs (29–32), providing a possible solution to the ethical objections that have obstructed the use of human ESCs in many countries. Similar to ESCs, iPSCs share genetic and transcriptomic signatures with cancer cells (14). Human iPSCs were first generated by the transduction of fibroblasts with four transcription factors: *OCT4, SOX2*, c-*MYC*, and *KLF4* (28). *C-MYC* is a well-known oncogene (33, 34), and the other three factors are also known to be upregulated in multiple cancers types (35–40). Indeed, one study showed significant overexpression of at least one of these factors in 18 of the 40 cancer types that were evaluated (41). Also, these genes are associated with tumor progression and poor prognosis in certain tumor types (41), suggesting that targeting these genes in cancers may be therapeutically beneficial.

A recent study analyzed and compared the epigenomic and transcriptomic signatures of human tumors from The Cancer Genome Atlas (TCGA) and ESCs, as well as iPSCs and other progenitor cells from Progenitor Cell Biology Consortium (PCBC) (42). In this study, the authors applied machine learning algorithms to reveal a positive correlation between tumor dedifferentiation status and stemness indices for most of the tumor cases they analyzed (42). Importantly, they also demonstrated that the cancer stemness indices are higher in recurrent and metastatic tumors than primary tumors, supporting the concept that cancer stem cells play essential roles in cancer recurrence and metastasis (43, 44). In addition, using single-cell transcriptome analysis the authors identified a heterogeneous expression of stemness-associated markers in patient tumors, suggesting the need for multi-target strategies when targeting cancer stem cells.

## Immunogenicity of ESCS and iPSCs

Embryonic stem cells are usually obtained from an unrelated donor due to their limited availability. Therefore, these cells often express mismatched major histocompatibility complex (MHC) and/or minor histocompatibility (miH) antigens and will trigger alloimmune responses when transplanted in the host. ESCs express low levels of HLA class I molecules (45) and almost undetectable levels of HLA class II and costimulatory molecules (46). Although expressed at a low level, HLA class I molecules in ESCs are sufficient to trigger xenorejection of human ESCs mediated by cytotoxic T cells (47, 48). ESCs induce potent humoral and cellular immune responses, leading to the infiltration of inflammatory cells that is followed by ESC rejection (49). So far, most immunogenicity studies of ESCs have focused on a scenario that involves MHC mismatches, implicating alloimmunity as one of the main players in the immune responses after ESCs transplantation. However, whether embryonic antigens in ESCs could induce an immune response is less clear.

Induced pluripotent stem cells are somatic cells that were reprogramed back to a pluripotent state. Autologous iPSCs can be generated from the person receiving therapy. Since the initial discovery of iPSCs, researchers immediately assumed that these cells would be a potential cell source of autologous cell-based therapies to bypass the issues of alloimmunity caused by allogeneic sources such as human ESCs or donated tissue (50, 51). However, later studies investigating iPSC immunogenicity in autologous settings raised questions about this assumption. Araki et al. (52) showed that autologous iPSC-derived teratomas were rejected by immune-competent mice and found a comparable level of rejection of autologous ESC-derived teratomas. These data suggest that in autologous transplantation models with minimized alloimmunity, other antigens such as embryonic antigens in ESCs and iPSCs could still induce an immune response. In 2014, we noticed that autologous iPSCs are immunogenic (11), contradicting earlier studies claiming they are immune privileged. We showed in murine models that undifferentiated autologous iPSCs elicited an immune response with increased lymphocytic infiltration and elevated granzyme-B, IFN-γ, and perforin intragraft. In contrast, autologous iPSC-derived endothelial cells were accepted by immune mechanisms similar to self-tolerance. These studies suggest that undifferentiated autologous iPSCs may express antigens of embryonic origin that can trigger an immune response, whereas fully differentiated cells derived from iPSCs have lower levels of immunogenicity. Based on these data and the similarity between iPSCs and cancer cells, we reached the conclusion that undifferentiated iPSCs are immunogenic and hypothesized that they can be used as a cancer vaccine.

## Oncofetal Peptide Vaccines and Whole-Cell Vaccines

### Oncofetal Peptide-Based Vaccines

A wide range of vaccines based on the aforementioned oncofetal antigens have been tested in pre-clinical studies, and some single antigen vaccines have been tested in clinical trials. Among all oncofetal antigens, many well-studied ones belong to a class of proteins called cancer testis antigens (CTAs) (Supplemental Table 1). CTAs are expressed within the immune-privileged environment of the testes as well as by tumor cells. Targeting CTAs can induce highly tumor-specific immune responses and thus provide an ideal strategy for anti-cancer vaccines. For example, a series of clinical trials have evaluated the CTA melanoma-specific antigen A3 (MAGE-A3) as a cancer vaccine target. MAGE-A3 is highly expressed in many different tumor types (53, 54). An early phase clinical trial demonstrated that adjuvant-mixed, recombinant MAGE-A3 proteins or peptide vaccines could elicit potent anti-tumor T cell and antibody responses which are associated with objective responses (54). However, a phase III trial in non-small-cell lung carcinoma (NSCLC) evaluating MAGE-A3 as an adjuvant treatment demonstrated no significant improvement in disease-free survival compared with placebo in MAGE-A3-positive patients. So far, no further clinical trials testing the MAGE-A3 targeting immunotherapies in NSCLC have been approved based on these results (55).

Another example of a single-peptide-antigen vaccine in clinical trial targeting glypican-3 taught us a similar lesson (56). In this phase II clinical trial, the investigators observed that two patients had tumor relapse despite significant numbers of vaccine-induced peptide-specific CTLs in their blood. Interestingly, they found that although glypican-3 was expressed in the primary tumor, the recurrent tumors lost the antigen expression. The investigators concluded that “the peptide vaccine may eradicate tumor cells that express such antigen, [and] cancer cells that do not express or lose the same antigen may then proliferate. In such cases, vaccines that target multiple shared antigens would be effective.”

Upon learning the lessons from failed early clinical trials using single-peptide cancer vaccines, later clinical trials evaluating peptide antigen-based cancer vaccines have focused mostly on multiple-peptide and antigens and/or are administered in combination with immunostimulatory adjuvants and other targeted therapies (57).

These results indicate that targeting one antigen alone may not be able to generate a sufficiently effective and durable anti-tumor immune response to mediate tumor rejection because of tumor heterogeneity and the rapid appearance of escape mutants. Therefore, it has been suggested that strategies that could target multiple tumor-associated antigens at once would induce a broader spectrum of anti-tumor immunity and possibly provide more effective and durable protection against cancer.

### ESC-Based Whole-Cell Cancer Vaccines

Since the establishment and characterization of human ESC lines, researchers have attempted to evaluate ESC-based whole-cell cancer vaccines due to their ability to deliver multiple oncofetal antigens in one treatment. In addition, unlike defined antigen-based vaccines, the whole-cell vaccine is universally applicable to all patients regardless of their HLA type. Li et al. found that human ESCs were able to induce a moderate anti-tumor effect (16). Both humoral and cellular immunity were activated by H9 ESC line, as evidenced by the production of colon carcinoma cell line-specific antibodies and IFNγ-producing cells, respectively. It was speculated that oncofetal antigens shared by the ESCs and tumors might have contributed to the vaccine-induced anti-tumor response. However, these immune responses were induced by a xenogeneic human ESC line injected into mice, and it is very likely that the incompatibility of the MHC antigens between the human ESCs and mouse cells contributed to a large portion of the immune responses. Furthermore, the anti-tumor effects produced by the xenogeneic ESC-vaccine were not as potent as those induced by immunization with the syngeneic murine colon cancer cells. A similar approach using xenogeneic human ESCs as a cancer prevention vaccine was evaluated by Zhang et al. (58) in an ovarian cancer model in rats, and a moderate tumor prevention effect was observed in this study.

These results raise the question of whether allogeneic or autologous ESCs are better than xenogeneic ESCs as an anti-cancer vaccine. A later study by Dong et al. (59) evaluated an allogeneic ESC cancer vaccine in mice. They investigated the ESC vaccine both as a prophylactic vaccine and as a therapeutic treatment in a transplantable lung cancer model by showing it could inhibit tumor growth in mice by enhancing lymphocyte proliferation and cytokine secretion, suggesting the potential of utilizing allogeneic ESC vaccines as a therapeutic strategy. However, they observed a stronger tumor inhibitory effect in the prophylactic group compared with the therapeutic group, which may be due to the immunosuppressive environment in established tumors.

To test the prophylactic ESC cancer vaccine in a physiologically relevant setting, Yaddanapudi et al. (60) employed a spontaneous mouse tumor model. Allogenic ESCs along with GM-CSF were used to provide immunostimulatory adjuvant activity. GM-CSF can stimulate and activate antigen-presenting cells (APCs), which can process and present tumor antigens to CD4+ helper T cells and CD8+ cytotoxic T lymphocytes (CTL) (61, 62). The authors observed more potent and durable protection against tumor growth than that found in earlier studies using ESCs alone, corroborating the immunostimulatory effects of the GM-CSF in the cancer vaccine. Moreover, this combinatory vaccination could inhibit carcinogen and chronic pulmonary inflammation induced lung cancer, which is a physiologically relevant spontaneous lung cancer model in mice.

### iPSC-Based Whole-Cell Cancer Vaccines

Embryonic stem cells and iPSCs share nearly identical gene expression and epigenetic profiles (29–32). Based on the similarities between cancer cells and ESCs, Li et al. (16) evaluated one human iPSC line TZ1 as an anti-cancer vaccine in a transplantable mouse colon cancer model. They found that although these iPSCs induced significant numbers of IFNγ- and IL-4-producing splenocytes against the mouse colon cancer cells, no evidence of tumor rejection was seen, possibly due to the accumulation of myeloid-derived suppressor cells in TZ1-immunized groups. These data suggest that modifications of the iPSC-based cancer vaccine are needed to increase the immune response against tumors. For example, autologous iPSCs may contain a more representative and accurate panel of tumor antigens than xenogeneic iPSCs, and therefore, autologous iPSCs may be better than xenogeneic iPSCs as anti-cancer vaccines, pending further confirmatory studies. In addition, an immunostimulatory vaccine adjuvant may enhance the anti-tumor immunity of the iPSC-based vaccines.

**Figure 1:**
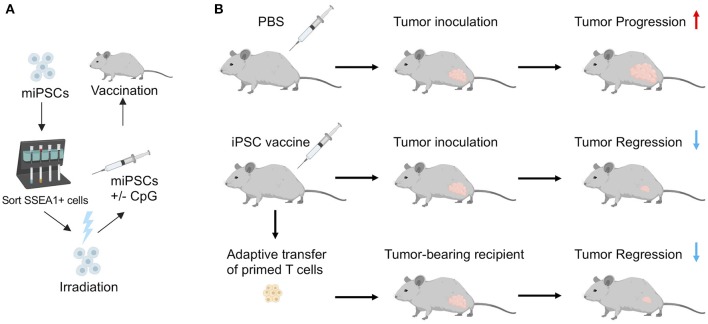
**(A)** Schematic illustration of vaccine preparation consisting of sorting murine iPSCs for a pluripotent marker, irradiation, resuspension in adjuvant solution (CpG), and subcutaneous injection in mice. **(B)** In a prophylactic setting, autologous iPSC vaccines prevent tumor growth in syngeneic murine models. Adoptive transfer of T cells isolated from vaccine-treated mice inhibited tumor growth in unvaccinated tumor-bearing recipients, indicating that the iPSC vaccine promotes an antigen-specific anti-tumor T cell response. Adapted from Kooreman et al. (14) with permission from Elsevier.

Embryonic/fetal materials or ESCs often come from unrelated donors and may express mismatched MHC that could trigger an immune response. To study the immunogenicity of oncofetal proteins, alloimmunity stimulated by MHC mismatches will need to be eliminated. In addition, tumorigenicity associated with ESCs has been one of the major obstacles in using ESCs as cancer vaccines for clinical applications. Recently, a study by our lab (14) addressed these issues using an irradiated autologous iPSC-based cancer vaccine. In this study, we first demonstrated that human and murine iPSCs express a list of tumor-associated and tumor-specific antigens by comparing expression profiles of 11 different human iPSC clones with human ESCs, cancer tissues, and healthy tissues using RNA sequencing. We showed that human iPSCs cluster with human ESCs and the cancer tissues, revealing significant gene expression overlap in cancer genes among different cancer types and iPSCs. To evaluate whether the oncofetal antigens in iPSCs rather than MHC mismatches could induce immune responses, we minimized alloimmunity by utilizing autologous iPSCs as the source of the anti-cancer vaccine. To enhance the anti-tumor immunity induced by the vaccine, we included an immunostimulatory adjuvant, CpG oligodeoxynucleotide, a toll-like receptor 9 (TLR 9) agonist that can induce the maturation of APCs (Figure 1). We then irradiated iPSCs before vaccination to prevent teratoma formation, as studies have shown that gamma irradiation could inhibit the tumorigenicity of iPSCs (63, 64). We irradiated iPSCs at 60 Gy, which is a lethal dose to human iPSCs *in vitro* and known to significantly decrease teratoma formation ability of human iPSCs in mice (63, 64). We generated autologous iPSCs by introducing Yamanaka factors (Oct4, Sox2, Klf4, and c-Myc) into mouse fibroblasts from the same mouse strain. Vaccinations with irradiated iPSCs mixed with the immunostimulatory CpG were administered weekly for a month, inducing antibodies that bound to iPSCs and tumor cells. Vaccination with iPSC-based cancer vaccine also induced CD4+ and CD8+ T cells that could recognize tumor cells *in vitro*, suggesting the induced immune responses are tumor specific. Vaccination increased APCs and activated T cells in mice, resulting in a favorable ratio of CD8+ T cells over CD4+CD25+FoxP3+ regulatory T cells (T-regs). As a result, vaccinated mice rejected transplanted breast cancer, melanoma, and mesothelioma tumor cells, indicating that the stimulated immune activity was tumor-specific and functional. Importantly, adoptive transfer of T cells isolated from vaccine-treated tumors could transfer this tumor protection to naïve mice, proving that the tumor protection effect was mediated by T cells (Figure 1).

Because preventive treatment of cancer is clinically uncommon for non-viral associated cancers, we also investigated the therapeutic effects of the iPSC-vaccine in established tumors. Here, the vaccination with iPSC vaccine did not stop the growth of established melanomas, which may be due to the established immunosuppressive tumor microenvironment. We then examined a clinically relevant scenario involving the surgical removal of the majority of tumors but left some residual tumor remains at the margins; we found that the iPSC + CpG vaccine could inhibit tumor relapse. These data are consistent with the finding that cancer stemness features are more highly expressed in recurrent tumors (42).

Because adult stem cells are also present, although rare, in some adult organs such as skin, liver, bone marrow, and digestive system (65), we evaluated auto-immunity by monitoring the animal body weight, organ histology, and antinuclear antibody levels. All of these measurements were normal, suggesting the absence of gross toxicity and autoimmunity in vaccinated mice. The iPSC vaccine could break the self-tolerance of the immune system to oncofetal antigens yet did not induce significant auto-immunity, which was possibly due to the higher abundance of these oncofetal antigens in tumors than in resident stem cells within organs. Taken together, our data support further assessing the value of iPSC-based whole-cell therapy as an anti-cancer immunotherapy.

## Concluding Remarks

Oncofetal antigen-based cancer vaccines have demonstrated therapeutic potential in preclinical and some clinical studies. As presented by several examples in this review, various oncofetal antigen-based vaccine strategies, particularly approaches that combine an autologous iPSC vaccine with an immune adjuvant, have demonstrated great promise to elicit potent anti-tumor responses for cancer treatment. Despite these advances, challenges remain. For instance, many early clinical studies using oncofetal antigen-based vaccines focused on single oncofetal antigens with or without immune adjuvants, limiting the level, and duration of the induced anti-tumor immune response due to tumor heterogenicity and fast adaptation of cancer cells. Unlike the defined antigen-based vaccines, whole-cell vaccines are universally applicable to all patients without concerns on HLA type mismatches. Therefore, whole cell-based cancer vaccines, with the epitope heterogeneity of wholes cells of ESCs and iPSCs, may prove more potent, durable, and easier to apply than single-antigen targeted vaccines.

The only FDA approved non-antiviral cancer vaccine, Sipuleucel-T (Provenge), was developed as a TAA pulsed autologous dendritic cell-based cancer vaccination for prostate cancer (66). In 2010, it was approved as an autologous whole-cell cancer vaccine that utilizes a TAA and GM-CSF fusion protein pulsed autologous peripheral blood mononuclear cells (PBMCs). It prolonged patient survival rate by 50% at 3 years in a phase III study, thus has been approved for treating patients with castration -resistant metastatic prostate cancer (67), supporting the efficacy of TAA-based cancer vaccine and the feasibility of using autologous whole-cell cancer vaccine in clinical settings.

**Figure 2:**
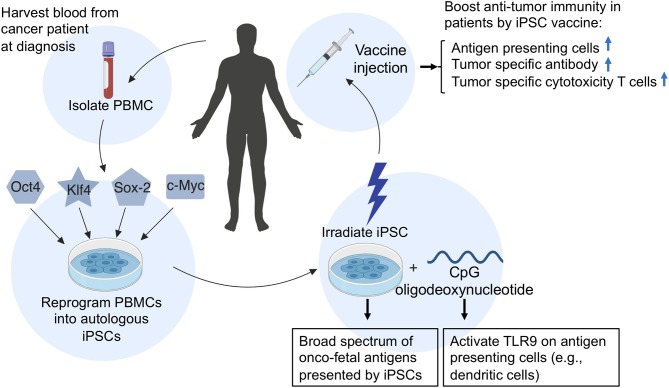
A schematic illustration of the generation and application of an autologous iPSC-based cancer vaccine in patients. To generate an autologous iPSC-based cancer vaccine, peripheral blood mononuclear cells (PBMCs) are isolated from the patient's blood and reprogrammed into induced pluripotent stem cells (iPSCs) by the introduction of four Yamanaka factors (Oct4, Klf4, Sox2, and c-Myc). The resulting patient-derived iPSCs are then irradiated and prepared in combination with CpG oligodeoxynucleotides and injected into patients as an anti-cancer vaccine. Upon vaccination of iPSC-autologous vaccine in patients, the irradiated iPSCs will provide a broad spectrum of oncofetal antigens, while CpGs will activate toll-like receptor 9 (TLR9) on antigen-presenting cells such as dendritic cells, B cells, and macrophages, which can process and present oncofetal antigens to helper T cells and cytotoxic T lymphocytes, thus conferring anti-tumor immunity.

In addition, because autologous iPSC-based cancer vaccines are relatively easy to generate (Figure 2), iPSC vaccines can be made available at short notice after a diagnosis, ready to be dispensed soon after surgery, chemotherapy, or radiation therapy when cancer cells are most vulnerable. Vaccination of iPSC-vaccines at this time could prime the immune system to target a broad spectrum of cancer-specific antigens to prevent recurrence of cancer, because recurrent and metastatic tumors have a higher level of stemness phenotype (42).

Concerns such as teratoma formation and auto-immunity must be addressed in evaluating the use of iPSC-based cancer vaccines in humans. Although the iPSC-based cancer vaccine did not induce significant auto-immunity in mice and injection of irradiated miPSCs did not result in teratoma formation in mice (14), differences in mouse and human iPSCs and immune systems should be carefully considered before moving this treatment to the clinical settings.

Approaches to further enhance the efficacy of iPSC-based cancer vaccines include concurrent treatment with PD-1/CTLA-4 checkpoint inhibitors, chemotherapy, or radiation therapy. Additional approaches include immunostimulatory agents that can more potently activate APCs, including agonistic CD40 monoclonal antibodies and other TLR agonists such as PolyI:C. These approaches offer powerful combination therapies with possible synergistic effects that may be more effective in patients who have a high risk of disease recurrence after receiving initial standard-of-care therapy.

## Author Contributions

XO wrote the manuscript. XO, MT, and JW revised the manuscript and provided the critical input.

### Conflict of Interest Statement

JW is the co-founder of Khloris Biosciences. However, research in his laboratory is independent from and not supported by Khloris Biosciences. The remaining authors declare that the research was conducted in the absence of any commercial or financial relationships that could be construed as a potential conflict of interest.

## References

[1] Chism SE Burton RC Warner NL. Immunogenicity of oncofetal antigens: a review. Clin Immunol Immunopathol. (1978) 11:346–73. 10.1016/0090-1229(78)90059-4 81731

[2] Trojan J Naval X Johnson T Lafarge-Frayssinet C Hajeri-Germond M Farges O. Expression of serum albumin and of alphafetoprotein in murine normal and neoplastic primitive embryonic structures. Mol Reprod Dev. (1995) 42:369–78. 10.1002/mrd.1080420402 8607965

[3] Haynes WDG Shertock KL Skinner JM Whitehead R. The ultrastructural immunohistochemistry of oncofoetal antigens in large bowel carcinomas. Virchows Arch A. (1985) 405:263–75. 10.1007/BF00704377 3918390

[4] Matzuk MM Krieger M Corless CL Boime I. Effects of preventing O-glycosylation on the secretion of human chorionic gonadotropin in Chinese hamster ovary cells. Proc Natl Acad Sci USA. (1987) 84:6354–8. 10.1073/pnas.84.18.6354 3476951 PMC299074

[5] Fishman WH Raam S Stolbach LL. Markers for ovarian cancer: regan isoenzyme and other glycoproteins. Semin Oncol. (1975)2:211–6. 63996

[6] Slodkowska J Szturmowicz M Rudzinski P Giedronowicz D Sakowicz A Androsiuk W. Expression of CEA and trophoblastic cell markers by lung carcinoma in association with histological characteristics and serum marker levels. Eur J Cancer Prev Off J Eur Cancer Prev Organ ECP. (1998) 7:51–60. 9511851

[7] Tran L Judor J-P Gauttier V Geist M Hoffman C Rooke R. The immunogenicity of the tumor-associated antigen α-fetoprotein is enhanced by a fusion with a transmembrane domain. BioMed Res Inter. (2012). 2012:878657. 10.1155/2012/878657 22500109 PMC3304459

[8] Skinner JM Whitehead R. Tumor-associated antigens in polyps and carcinoma of the human large bowel. Cancer. (1981) 47:1241–5. 10.1002/1097-0142(19810315)47:6<1241::AID-CNCR2820470602>3.0.CO;2-Y 6164469

[9] Purswani S Talwar GP. Development of a highly immunogenic recombinant candidate vaccine against human chorionic gonadotropin. Vaccine. (2011) 29:2341–8. 10.1016/j.vaccine.2010.11.069 21272600

[10] Takahashi K Yamanaka S. Induction of pluripotent stem cells from mouse embryonic and adult fibroblast cultures by defined factors. Cell. (2006) 126:663–76. 10.1016/j.cell.2006.07.024 16904174

[11] de Almeida PE Meyer EH Kooreman NG Diecke S Dey D Sanchez-Freire V. Transplanted terminally differentiated induced pluripotent stem cells are accepted by immune mechanisms similar to self-tolerance. Nat Commun. (2014) 5:3903. 10.1038/ncomms4903 24875164 PMC4075468

[12] Ghosh Z Huang M Hu S Wilson KD Dey D Wu JC Dissecting the oncogenic potential of human embryonic and induced pluripotent stem cell derivatives. Cancer Res. (2011) 71:5030–9. 10.1158/0008-5472.CAN-10-4402 21646469 PMC3138859

[13] Brewer BG Mitchell RA Harandi A Eaton JW. Embryonic vaccines against cancer: an early history. Exp Mol Pathol. (2009) 86:192–7. 10.1016/j.yexmp.2008.12.002 19171137

[14] Kooreman NG Kim Y de Almeida PE Termglinchan V Diecke S Shao N-Y. Autologous iPSC-based vaccines elicit anti-tumor responses *in vivo*. Cell Stem Cell. (2018) 22:501–13.e7. 10.1016/j.stem.2018.01.016 29456158 PMC6134179

[15] Zheng Q Zheng Y Chen J You J Zhu Y Liu Y. A hepatic stem cell vaccine is superior to an embryonic stem cell vaccine in the prophylaxis and treatment of murine hepatocarcinoma. Oncol Rep. (2017) 37:1716–24. 10.3892/or.2017.5381 28098898

[16] Li Y Zeng H Xu R-H Liu B Li Z. Vaccination with human pluripotent stem cells generates a broad spectrum of immunological and clinical responses against colon cancer. Stem Cells Dayt Ohio. (2009) 27:3103–11. 10.1002/stem.234 19816950

[17] Ben-David U Benvenisty N. The tumorigenicity of human embryonic and induced pluripotent stem cells. Nat Rev Cancer. (2011) 11:268–77. 10.1038/nrc3034 21390058

[18] Hiyama E Hiyama K. Telomere and telomerase in stem cells. Br J Cancer. (2007) 96:1020–4. 10.1038/sj.bjc.6603671 17353922 PMC2360127

[19] Heisig J Weber D Englberger E Winkler A Kneitz S Sung W-K. Target Gene analysis by microarrays and chromatin immunoprecipitation identifies HEY proteins as highly redundant bHLH repressors. PLoS Genet. (2012) 8:e1002728. 10.1371/journal.pgen.1002728 22615585 PMC3355086

[20] Evans PM Liu C. Roles of Krüpel-like factor 4 in normal homeostasis, cancer and stem cells. Acta Biochim Biophys Sin. (2008) 40:554–64. 10.1111/j.1745-7270.2008.00439.x 18604447 PMC2668950

[21] Sperger JM Chen X Draper JS Antosiewicz JE Chon CH Jones SB. Gene expression patterns in human embryonic stem cells and human pluripotent germ cell tumors. Proc Natl Acad Sci USA. (2003) 100:13350–5. 10.1073/pnas.2235735100 14595015 PMC263817

[22] Ben-Porath I Thomson MW Carey VJ Ge R Bell GW Regev A. An embryonic stem cell-like gene expression signature in poorly differentiated aggressive human tumors. Nat Genet. (2008) 40:499–507. 10.1038/ng.127 18443585 PMC2912221

[23] Neveu P Kye MJ Qi S Buchholz DE Clegg DO Sahin M. MicroRNA profiling reveals two distinct p53-related human pluripotent stem cell states. Cell Stem Cell. (2010) 7:671–81. 10.1016/j.stem.2010.11.012 21112562

[24] Calvanese V Horrillo A Hmadcha A Suarez-Alvarez B Fernandez AF Lara E. Cancer genes hypermethylated in human embryonic stem cells. PloS ONE. (2008) 3:e3294. 10.1371/journal.pone.0003294 18820729 PMC2546447

[25] Baker DEC Harrison NJ Maltby E Smith K Moore HD Shaw PJ. Adaptation to culture of human embryonic stem cells and oncogenesis *in vivo*. Nat Biotechnol. (2007) 25:207–15. 10.1038/nbt1285 17287758

[26] Thomson JA Itskovitz-Eldor J Shapiro SS Waknitz MA Swiergiel JJ Marshall VS. Embryonic stem cell lines derived from human blastocysts. Science. (1998) 282:1145–7. 10.1126/science.282.5391.1145 9804556

[27] Hanahan D Weinberg RA. Hallmarks of cancer: the next generation. Cell. (2011) 144:646–74. 10.1016/j.cell.2011.02.013 21376230

[28] Takahashi K Tanabe K Ohnuki M Narita M Ichisaka T Tomoda K. Induction of pluripotent stem cells from adult human fibroblasts by defined factors. Cell. (2007) 131:861–72. 10.1016/j.cell.2007.11.019 18035408

[29] Bock C Kiskinis E Verstappen G Gu H Boulting G Smith ZD. Reference maps of human ES and iPS cell variation enable high-throughput characterization of pluripotent cell lines. Cell. (2011) 144:439–52. 10.1016/j.cell.2010.12.032 21295703 PMC3063454

[30] Mallon BS Hamilton RS Kozhich OA Johnson KR Fann YC Rao MS. Comparison of the molecular profiles of human embryonic and induced pluripotent stem cells of isogenic origin. Stem Cell Res. (2014) 12:376–86. 10.1016/j.scr.2013.11.010 24374290 PMC4157340

[31] Mallon BS Chenoweth JG Johnson KR Hamilton RS Tesar PJ Yavatkar AS. StemCellDB: the human pluripotent stem cell database at the national institutes of health. Stem Cell Res. (2013) 10:57–66. 10.1016/j.scr.2012.09.002 23117585 PMC3590800

[32] Soldner F Hockemeyer D Beard C Gao Q Bell GW Cook EG. Parkinson's disease patient-derived induced pluripotent stem cells free of viral reprogramming factors. Cell. (2009) 136:964–77. 10.1016/j.cell.2009.02.013 19269371 PMC2787236

[33] Albihn A Johnsen JI Henriksson MA. MYC in oncogenesis and as a target for cancer therapies. Adv Cancer Res. (2010) 107:163–224. 10.1016/S0065-230X(10)07006-5 20399964

[34] Ruggero D. The role of Myc-induced protein synthesis in cancer. Cancer Res. (2009) 69:8839–43. 10.1158/0008-5472.CAN-09-1970 19934336 PMC2880919

[35] Tian Y Luo A Cai Y Su Q Ding F Chen H. MicroRNA-10b promotes migration and invasion through KLF4 in human esophageal cancer cell lines. J Biol Chem. (2010) 285:7986–94. 10.1074/jbc.M109.062877 20075075 PMC2832949

[36] Lambertini C Pantano S Dotto GP. Differential control of Notch1 gene transcription by Klf4 and Sp3 transcription factors in normal versus cancer-derived keratinocytes. PloS ONE. (2010) 5:e10369. 10.1371/journal.pone.0010369 20442780 PMC2860992

[37] Rageul J Mottier S Jarry A Shah Y Théoleyre S Masson D. KLF4-dependent, PPARgamma-induced expression of GPA33 in colon cancer cell lines. Int J Cancer. (2009) 125:2802–9. 10.1002/ijc.24683 19551868 PMC2791338

[38] Asadi MH Mowla SJ Fathi F Aleyasin A Asadzadeh J Atlasi Y. OCT4B1, a novel spliced variant of OCT4, is highly expressed in gastric cancer and acts as an antiapoptotic factor. Int J Cancer. (2011) 128:2645–52. 10.1002/ijc.25643 20824712

[39] Ji J Zheng P-S. Expression of Sox2 in human cervical carcinogenesis. Hum Pathol. (2010) 41:1438–47. 10.1016/j.humpath.2009.11.021 20709360

[40] Sholl LM Barletta JA Yeap BY Chirieac LR Hornick JL. Sox2 protein expression is an independent poor prognostic indicator in stage I lung adenocarcinoma. Am J Surg Pathol. (2010) 34:1193–8. 10.1097/PAS.0b013e3181e5e024 20631605 PMC2923819

[41] Schoenhals M Kassambara A De Vos J Hose D Moreaux J Klein B. Embryonic stem cell markers expression in cancers. Biochem Biophys Res Commun. (2009) 383:157–62. 10.1016/j.bbrc.2009.02.156 19268426

[42] Malta TM Sokolov A Gentles AJ Burzykowski T Poisson L Weinstein JN. Machine learning identifies stemness features associated with oncogenic dedifferentiation. Cell. (2018) 173:338-354.e15. 10.1016/j.cell.2018.03.034 29625051 PMC5902191

[43] Clarke MF. Clinical and therapeutic implications of cancer stem cells. N Engl J Med. (2019) 380:2237–45. 10.1056/NEJMra1804280 31167052

[44] Shiozawa Y Nie B Pienta KJ Morgan TM Taichman RS. Cancer stem cells and their role in metastasis. Pharmacol Ther. (2013) 138:285–93. 10.1016/j.pharmthera.2013.01.014 23384596 PMC3602306

[45] Drukker M Katz G Urbach A Schuldiner M Markel G Itskovitz-Eldor J. Characterization of the expression of MHC proteins in human embryonic stem cells. Proc Natl Acad Sci USA. (2002) 99:9864–9. 10.1073/pnas.142298299 12114532 PMC125045

[46] Drukker M Katchman H Katz G Even-Tov Friedman S Shezen E Hornstein E. Human embryonic stem cells and their differentiated derivatives are less susceptible to immune rejection than adult cells. Stem Cells Dayt Ohio. (2006) 24:221–9. 10.1634/stemcells.2005-0188 16109762

[47] Robertson NJ Brook FA Gardner RL Cobbold SP Waldmann H Fairchild PJ. Embryonic stem cell-derived tissues are immunogenic but their inherent immune privilege promotes the induction of tolerance. Proc Natl Acad Sci USA. (2007) 104:20920–5. 10.1073/pnas.0710265105 18093946 PMC2409242

[48] Bifari F Pacelli L Krampera M. Immunological properties of embryonic and adult stem cells. World J Stem Cells. (2010) 2:50–60. 10.4252/wjsc.v2.i3.50 21607122 PMC3097923

[49] Grinnemo K-H Kumagai-Braesch M Månsson-Broberg A Skottman H Hao X Siddiqui A. Human embryonic stem cells are immunogenic in allogeneic and xenogeneic settings. Reprod Biomed Online. (2006) 13:712–24. 10.1016/S1472-6483(10)60663-3 17169186

[50] Nishikawa S Goldstein RA Nierras CR. The promise of human induced pluripotent stem cells for research and therapy. Nat Rev Mol Cell Biol. (2008) 9:725–9. 10.1038/nrm2466 18698329

[51] Zhao R Daley GQ. From fibroblasts to iPS cells: induced pluripotency by defined factors. J Cell Biochem. (2008) 105:949–55. 10.1002/jcb.21871 18668528

[52] Araki R Uda M Hoki Y Sunayama M Nakamura M Ando S Negligible immunogenicity of terminally differentiated cells derived from induced pluripotent or embryonic stem cells. Nature. (2013) 494:100–4. 10.1038/nature11807 23302801

[53] Brichard VG Lejeune D. GSK's antigen-specific cancer immunotherapy programme: pilot results leading to Phase III clinical development. Vaccine. (2007) 25(Suppl 2):B61–71. 10.1016/j.vaccine.2007.06.038 17916463

[54] Atanackovic D Altorki NK Cao Y Ritter E Ferrara CA Ritter G. Booster vaccination of cancer patients with MAGE-A3 protein reveals long-term immunological memory or tolerance depending on priming. Proc Natl Acad Sci USA. (2008) 105:1650–5. 10.1073/pnas.0707140104 18216244 PMC2234199

[55] Vansteenkiste JF Cho BC Vanakesa T De Pas T Zielinski M Kim MS. Efficacy of the MAGE-A3 cancer immunotherapeutic as adjuvant therapy in patients with resected MAGE-A3-positive non-small-cell lung cancer (MAGRIT): a randomised, double-blind, placebo-controlled, phase 3 trial. Lancet Oncol. (2016) 17:822–35. 10.1016/S1470-2045(16)00099-1 27132212

[56] Shimizu Y Suzuki T Yoshikawa T Tsuchiya N Sawada Y Endo I Cancer immunotherapy-targeted glypican-3 or neoantigens. Cancer Sci. (2018) 109:531–41. 10.1111/cas.13485 29285841 PMC5834776

[57] Bezu L Kepp O Cerrato G Pol J Fucikova J Spisek R. Trial watch: peptide-based vaccines in anticancer therapy. Oncoimmunology. (2018) 7:e1511506. 10.1080/2162402X.2018.1511506 30524907 PMC6279318

[58] Zhang Z-J Chen X-H Chang X-H Ye X Li Y Cui H. Human embryonic stem cells–a potential vaccine for ovarian cancer. Asian Pac J Cancer Prev APJCP. (2012) 13:4295–300. 10.7314/APJCP.2012.13.9.4295 23167331

[59] Dong W Du J Shen H Gao D Li Z Wang G. Administration of embryonic stem cells generates effective antitumor immunity in mice with minor and heavy tumor load. Cancer Immunol Immunother CII. (2010) 59:1697–705. 10.1007/s00262-010-0899-9 20683592 PMC11030618

[60] Yaddanapudi K Mitchell RA Putty K Willer S Sharma RK Yan J. Vaccination with embryonic stem cells protects against lung cancer: is a broad-spectrum prophylactic vaccine against cancer possible? PLoS ONE. (2012) 7:e42289. 10.1371/journal.pone.0042289 22860107 PMC3409174

[61] Nemunaitis J Sterman D Jablons D Smith JW Fox B Maples P. Granulocyte-macrophage colony-stimulating factor gene-modified autologous tumor vaccines in non-small-cell lung cancer. J Natl Cancer Inst. (2004) 96:326–31. 10.1093/jnci/djh028 14970281

[62] Dranoff G. GM-CSF-based cancer vaccines. Immunol Rev. (2002) 188:147–54. 10.1034/j.1600-065X.2002.18813.x 12445288

[63] Katsukawa M Nakajima Y Fukumoto A Doi D Takahashi J. Fail-safe therapy by gamma-ray irradiation against tumor formation by human-induced pluripotent stem cell-derived neural progenitors. Stem Cells Dev. (2016) 25:815–25. 10.1089/scd.2015.0394 27059007

[64] Inui S Minami K Ito E Imaizumi H Mori S Koizumi M. Irradiation strongly reduces tumorigenesis of human induced pluripotent stem cells. J Radiat Res (Tokyo). (2017) 58:430–8. 10.1093/jrr/rrw124 28340154 PMC5570064

[65] The Adult Stem Cell Stemcells.nih.gov. Available online at: https://stemcells.nih.gov/info/2001report/chapter4.htm (cited 2019 May 16).

[66] Fong L Brockstedt D Benike C Breen JK Strang G Ruegg CL. Dendritic cell-based xenoantigen vaccination for prostate cancer immunotherapy. J Immunol Baltim Md 1950. (2001) 167:7150–6. 10.4049/jimmunol.167.12.7150 11739538

[67] Cheever MA Higano CS. PROVENGE (Sipuleucel-T) in prostate cancer: the first FDA-approved therapeutic cancer vaccine. Clin Cancer Res. (2011) 17:3520–6. 10.1158/1078-0432.CCR-10-3126 21471425

[68] Grozdanov PN Yovchev MI Dabeva MD. The oncofetal protein glypican-3 is a novel marker of hepatic progenitor/oval cells. Lab Investig J Tech Methods Pathol. (2006) 86:1272–84. 10.1038/labinvest.3700479 17117158

[69] Slodkowska J Szturmowicz M Rudzinski P Giedronowicz D Sakowicz A Androsiuk W. Expression of CEA and trophoblastic cell markers by lung carcinoma in association with histological characteristics and serum marker levels. Eur J Cancer Prev Off J Eur Cancer Prev Organ ECP. (1998) 7:51–60. 9511851

[70] Baumhoer D Tornillo L Stadlmann S Roncalli M Diamantis EK Terracciano LM. Glypican 3 expression in human nonneoplastic, preneoplastic, and neoplastic tissues: a tissue microarray analysis of 4,387 tissue samples. Am J Clin Pathol. (2008) 129:899–906. 10.1309/HCQWPWD50XHD2DW6 18480006

[71] Coggin JH Adkinson L Anderson NG. Fetal antigens shared as transplantation rejection antigens on chemically induced mouse and hamster sarcomas. Cancer Res. (1980) 40:1568–73. 6768448

[72] Hishinuma M Ohashi K-I Yamauchi N Kashima T Uozaki H Ota S. Hepatocellular oncofetal protein, glypican 3 is a sensitive marker for alpha-fetoprotein-producing gastric carcinoma. Histopathology. (2006) 49:479–86. 10.1111/j.1365-2559.2006.02522.x 17064293

[73] Kato T Hayama S Yamabuki T Ishikawa N Miyamoto M Ito T. Increased expression of insulin-like growth factor-II messenger RNA-binding protein 1 is associated with tumor progression in patients with lung cancer. Clin Cancer Res Off J Am Assoc Cancer Res. (2007) 13(2 Pt 1):434–42. 10.1158/1078-0432.CCR-06-1297 17255263

[74] Shi YY Wang HC Yin YH Sun WS Li Y Zhang CQ. Identification and analysis of tumour-associated antigens in hepatocellular carcinoma. Br J Cancer. (2005) 92:929–34. 10.1038/sj.bjc.6602460 15756260 PMC2361901

[75] Jiang XP Yang DC Elliott RL Head JF. Vaccination with a mixed vaccine of autogenous and allogeneic breast cancer cells and tumor associated antigens CA15-3, CEA and CA125 - results in immune and clinical responses in breast cancer patients. Cancer Biother Radiopharm. (2000) 15:495–505. 10.1089/cbr.2000.15.495 11155821

[76] Coggin JH Rohrer JW Barsoum AL. True immunogenicity of oncofetal antigen/immature laminin receptor protein. Cancer Res. (2004) 64:4685. 10.1158/0008-5472.CAN-03-2940 15231682

[77] Fishman WH Raam S Stolbach LL. Markers for ovarian cancer: regan isoenzyme and other glycoproteins. Semin Oncol. (1975) 2:211–6. 63996

[78] Nouri AME Torabi-Pour N Dabare AANPM. A new highly specific monoclonal antibody against placental alkaline phosphatase: a potential marker for the early detection of testis tumour. BJU Int. (2000) 86:894–900. 10.1046/j.1464-410x.2000.00939.x 11069419

[79] Lüftl M Schuler G Jungbluth AA. Melanoma or not? Cancer testis antigens may help. Br J Dermatol. (2004) 151:1213–8. 10.1111/j.1365-2133.2004.06260.x 15606517

[80] Mizukoshi E Nakamoto Y Tsuji H Yamashita T Kaneko S. Identification of alpha-fetoprotein-derived peptides recognized by cytotoxic T lymphocytes in HLA-A24+ patients with hepatocellular carcinoma. Int J Cancer. (2006) 118:1194–204. 10.1002/ijc.21468 16152611

[81] Liu W Li Y Wang B Dai L Qian W Zhang J-Y. Autoimmune response to IGF2 mRNA-binding protein 2 (IMP2/p62) in breast cancer. Scand J Immunol. (2015) 81:502–7. 10.1111/sji.12285 25721883 PMC4431935

[82] Broe MED Pollet DE. Multicenter evaluation of human placental alkaline phosphatase as a possible tumor-associated antigen in serum. Clin Chem. (1988) 34:1995–9. 3168210

[83] Szmania S Gnjatic S Tricot G Stone K Zhan F Moreno A. Immunization with a recombinant MAGE-A3 protein after high-dose therapy for myeloma. J Immunother Hagerstown Md 1997. (2007) 30:847–54. 10.1097/CJI.0b013e318158fcff 18049337

[84] Malatesta M Mannello F Luchetti F Marcheggiani F Condemi L Papa S. Prostate-specific antigen synthesis and secretion by human placenta: a physiological kallikrein source during pregnancy. J Clin Endocrinol Metab. (2000) 85:317–21. 10.1210/jcem.85.1.6302 10634405

[85] Eifuku R Takenoyama M Yoshino I Imahayashi S So T Yasuda M. Analysis of MAGE-3 derived synthetic peptide as a human lung cancer antigen recognized by cytotoxic T lymphocytes. Int J Clin Oncol. (2001) 6:34–9. 10.1007/PL00012077 11706525

