# Supplementary material for: Induced Pluripotent Stem Cell-Based Cancer Vaccines
Source: Front Immunol. 2019 Jul 8;10:1510. doi: 10.3389/fimmu.2019.01510 (PMC6628907; doi:10.3389/fimmu.2019.01510)
Supplement: Supplementary file 1 [file Table_1.docx]

| **Supplemental Table 1 Oncofetal protein expression in different cancers and their immunogenicity** | | | | |
| --- | --- | --- | --- | --- |
| **Oncofetal Protein** | **Cancer** | **Immunogenicity** | **Expression** | **Reference** |
| α-fetoprotein (AFP) | Hepatocellular carcinoma, yolk sac tumor, colon and ovarian | AFP‐targeting immunotherapy could induce tumor-specific CTL | Produced by the yolk sac and the fetal liver during fetal development and by tumors. | (2,8,80) |
| Human chorionic gonadotropin (HCG) | Colon, ovarian, lung and prostate cancer | Not immunogenic | Synthesized and secreted during pregnancy and in tumors. | (8,77) |
| Glypican 3 | Hepatocellular carcinoma and melanoma | Glypican‐3 peptide vaccines induce specific CTLs in most patients | Highly expressed in normal ovarian, mammary, and mesothelial cells as well as in hepatocellular carcinoma, embryonal tumors, and colon cancer. | (68,70,72) |
| Cancer/testis antigen (CTA) | Melanoma, ovarian, lung, prostate, sarcoma and hepatocellular cancer | Immunogenic. It has been tested as cancer vaccines in clinical trials. | Expressed in various human cancers but, aside from the testicles, not in normal tissue. | (71,74,79,83-85) |
| Carcinoembryonic antigen (CEA) | Lung, colon, ovarian and breast cancer | When CEA-derived antigens were delivered as recombinant vectors, peptides, or full-length proteins, it induces variable delayed-type hypersensitivity responses in patients upon vaccination. | Overexpressed in colorectal, gastric, and pancreatic carcinomas, breast cancers, and non-small cell lung carcinomas and is expressed at lower levels in normal epithelial cells and fetal tissue. | (3,69,75) |
| Immature laminin receptor (OFA-iLR) | Breast, lung, ovary and prostate carcinoma, lymphoma and renal cell carcinoma | Immunogenic antigen in human renal cell carcinoma. OFA-iLR specific effector T cells producing interferon-gamma may have a role in the control of tumor growth. | Tumor-specific when expressed as a 37-kDa, nonacylated, monomeric protein in association with tumor cell plasma membranes. | (76) |
| Placental alkaline phosphatase (PLAP) | Colon, ovarian, breast, lung, testicular and gastric cancer | Specific antibody to PLAP is present in placental extracts, thus immunogenic in pregnancy. | Expressed on the syncy-tiotrophoblast of the placenta and in tumors. | (8,78,82) |
| IMP family (IGF2 mRNA-binding protein) | Non-small cell lung cancers and breast cancer. | Induces autoantibody in breast cancer | Expressed in embryonic tissues and a variety of cancers. | (73,81) |
